# Complete Biosynthesis of Anthocyanins Using *E. coli* Polycultures

**DOI:** 10.1128/mBio.00621-17

**Published:** 2017-06-06

**Authors:** J. Andrew Jones, Victoria R. Vernacchio, Shannon M. Collins, Abhijit N. Shirke, Yu Xiu, Jacob A. Englaender, Brady F. Cress, Catherine C. McCutcheon, Robert J. Linhardt, Richard A. Gross, Mattheos A. G. Koffas

**Affiliations:** a Department of Chemical and Biological Engineering, Rensselaer Polytechnic Institute, Troy, New York, USA; b Department of Biological Sciences, Rensselaer Polytechnic Institute, Troy, New York, USA; c Department of Chemistry, Rensselaer Polytechnic Institute, Troy, New York, USA; d Department of Chemistry, Hamilton College, Clinton, New York, USA; e State Key Laboratory of Chemical Resource Engineering, Beijing University of Chemical Technology, Beijing, China; f Beijing Key Laboratory of Bioactive Substances and Functional Food, Beijing Union University, Beijing, China; Korea Advanced Institute of Science and Technology

**Keywords:** *Escherichia coli*, anthocyanins, coculture, *de novo*, flavonoids, pelargonidin 3-*O*-glucoside, polyculture, recombinant production

## Abstract

Fermentation-based chemical production strategies provide a feasible route for the rapid, safe, and sustainable production of a wide variety of important chemical products, ranging from fuels to pharmaceuticals. These strategies have yet to find wide industrial utilization due to their inability to economically compete with traditional extraction and chemical production methods. Here, we engineer for the first time the complex microbial biosynthesis of an anthocyanin plant natural product, starting from sugar. This was accomplished through the development of a synthetic, 4-strain *Escherichia coli* polyculture collectively expressing 15 exogenous or modified pathway enzymes from diverse plants and other microbes. This synthetic consortium-based approach enables the functional expression and connection of lengthy pathways while effectively managing the accompanying metabolic burden. The *de novo* production of specific anthocyanin molecules, such as calistephin, has been an elusive metabolic engineering target for over a decade. The utilization of our polyculture strategy affords milligram-per-liter production titers. This study also lays the groundwork for significant advances in strain and process design toward the development of cost-competitive biochemical production hosts through nontraditional methodologies.

## IMPORTANCE

To efficiently express active extensive recombinant pathways with high flux in microbial hosts requires careful balance and allocation of metabolic resources such as ATP, reducing equivalents, and malonyl coenzyme A (malonyl-CoA), as well as various other pathway-dependent cofactors and precursors. To address this issue, we report the design, characterization, and implementation of the first synthetic 4-strain polyculture. Division of the overexpression of 15 enzymes and transcription factors over 4 independent strain modules allowed for the division of metabolic burden and for independent strain optimization for module-specific metabolite needs. This study represents the most complex synthetic consortia constructed to date for metabolic engineering applications and provides a new paradigm in metabolic engineering for the reconstitution of extensive metabolic pathways in nonnative hosts.

## INTRODUCTION

Microbial communities are ubiquitous in nature. In much the same way that multicellular eukaryotic organisms have evolved to contain specialized organelles that work together to seamlessly perform specialized tasks, communities of unicellular organisms have evolved similar divisions within their populations, such that microbial consortia amount to more than simply a sum of individual parts (1–5). These complex consortia allow for a cellular specialization, enabling the community to withstand larger environmental perturbations and perform more complex tasks than any of its individual constituents. Employment of this “division of labor” approach allows for a burden to be distributed across the population, permitting increased efficiency and more complex behavior than is possible in monoculture.

The study and application of natural microbial consortia have been topics of interest in the scientific literature for decades (6–8); however, the development of synthetic consortia, specifically consortia for metabolic engineering applications, has gained significant traction in the past few years (9–13). Several excellent examples of employing microbial communities for metabolic engineering have resulted in significant improvements over monoculture efforts (14). These gains were realized through utilization of the key advantages of microbial consortia, including (i) selection of the most efficient organism for the bioconversion (i.e., mixing bacterial and fungal hosts in a single consortium), (ii) use of traditional metabolic engineering principles (“push, pull, block”) to optimize each module for its specific cofactor and precursor needs, (iii) taking advantage of consortium modularity such that individual strains can be genetically optimized in monoculture and then applied in mixed culture without the need to reperform the genetic optimization, and (iv) capitalizing on the natural transportation of intermediate pathway metabolites in and out of the cells. The latter point could be considered a disadvantage of the polyculture strategy when transport does not occur naturally and is not easily engineered. Given the benefits of microbial consortia, however, this presents a unique motivation for researchers to vigorously investigate and engineer both natural and novel transport systems for targeted pathway intermediates.

**FIG 1:**
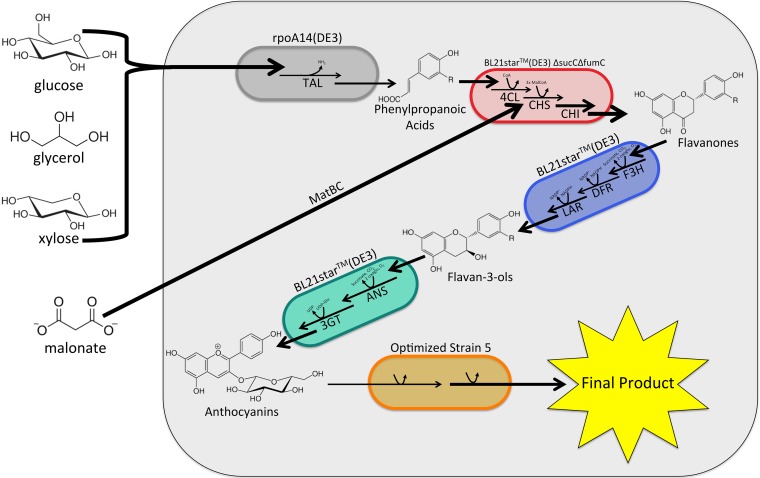
Polyculture schematic representing the realized 4-strain polyculture. Inclusion of a fifth strain shows the potential for extension through addition of sequential modules.

Although successful, these efforts have primarily focused on utilizing two strains in coculture. The development of polycultures, defined as the combination of three or more strains in a single coculture, is an important next step toward the goal of developing synthetic consortia that can rival the complexity of systems found in nature. In the present study, we discuss the development and application of a modular polyculture system for the production of high-value flavonoid products. Specifically, we leverage the power of microbial polycultures to demonstrate, for the first time, the *de novo* production of flavan-3-ols and anthocyanidin-3-*O*-glucosides in microbial hosts (Fig. 1). To accomplish this task, we built upon our previous coculture demonstration (9) by developing a phenylpropanoic acid production module capable of the highest-titer production of *p*-coumaric and caffeic acids to date. Combining this module with the previously developed C5 and p168 modules (9) enabled production of 26.1 mg/liter (+)-afzelechin from glucose. Finally, we further demonstrate the modularity of our system through production of anthocyanidin-3-*O-*glucosides from glucose by introduction of a fourth module for anthocyanin production to the system, resulting in a *de novo* titer of 9.5 ± 0.6 mg/liter pelargonidin-3-*O*-glucoside (callistephin), the predominant red pigment in strawberries (15). Critically, this production was obtained with only minimal fermentation optimization at the polyculture level. This work exemplifies the potential of mixed cultures to expand what is currently attainable for metabolic engineering applications and lays the groundwork for the development of more complex, higher-order polyculture communities capable of reconstituting extensive natural product pathways in nonnatural hosts.

## RESULTS

Expanding upon previous coculture efforts, the development of two additional bioconversion modules has been accomplished to realize the *de novo* production of both flavan-3-ols and anthocyanidin-3-*O-*glucosides for the first time outside of plants.

### Development of TAL module.

Significant efforts have been focused on improving the *de novo* production of phenylpropanoic acids in *Escherichia coli*. Similar endeavors have enabled production of flavan-3-ols and anthocyanins by supplementing phenylpropanoic acids or flavanones, but connection of the entire pathway in microbes has remained elusive (16, 17), possibly due to low outputs of the upstream pathway segments. Recent efforts from two labs have, however, enabled the near-gram-scale *de novo* production of both *p*-coumaric and caffeic acids (18, 19). The development of the tyrosine-overproducing *E. coli* strain rpoA14(DE3) represents a major milestone for the *de novo* production of phenylpropanoic acids (20, 21), while the discovery and optimization of the natively silenced *E. coli* non-P450 hydroxylase HpaBC enabled, for the first time, efficient production of caffeic acid through the ortho-hydroxylation of *p*-coumaric acid (18, 22–24). Building off of these efforts, we first set out to develop a phenylpropanoic acid production module that was compatible with our previously described “C5” and “p168” modules to enable the *de novo* production of flavan-3-ols *in vivo* (9).

**FIG 2:**
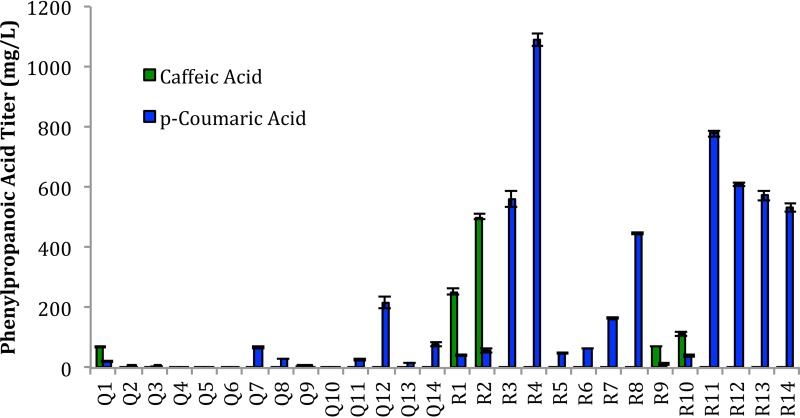
Screening of phenylpropanoic acid production modules. Initial screening was completed under optimal conditions for C5 and p168 coculture (9) (AMM–2% glycerol, 5-h induction point, 30°C fermentation temperature postinduction with 1 mM IPTG). Constitutive expression modules (Q11 to Q14 and R11 to R14) did not require induction with IPTG. The titers reported are after 2 days of cultivation in 48-well plates.

To accomplish this task, we collected the most efficient plasmids and strains from recent literature reports (18, 20, 25) and, along with several plasmids constructed in our lab, built 28 strain-plasmid combinations for screening of phenylpropanoic acid production (Table 1). Of the 28 strains, 20 were designed for *p*-coumaric acid production (tyrosine ammonia lyase [TAL] overexpression), while the remaining 8 were targeted for caffeic acid production (TAL and HpaBC overexpression). The effect of the endogenous gene supplementation plasmid pCS-TPTA was also tested but did not show significant titer improvements for any of the tested combinations (Fig. 2). This is in contrast with previous findings (18, 24); however, it is important to note that the screening was not completed under identical conditions. From the strain combinations, strain R4 represented the best *p*-coumaric acid producer, while strain R2 was selected as the best caffeic acid producer. It is interesting to note that neither R2 nor R4 represents a strain configuration that had been previously published, indicating that significant improvements can be attained through basic literature review and combinatorial screening of available modules.

**TABLE 1:** Phenylpropanoic acid production modules assessed in this work

Module^a^	Plasmid(s)
Q1 or R1	pZE-TH2, pCS-TPTA
Q2 or R2	pZE-TH2
Q3 or R3	pETM6-*Rg*TAL^syn^, pCS-TPTA
Q4 or R4	pETM6-*Rg*TAL^syn^
Q5 or R5	pCA1, pCS-TPTA
Q6 or R6	pCA1
Q7 or R7	pCA3, pCS-TPTA
Q8 or R8	pCA3
Q9 or R9	pETM6-*Rg*TAL^syn^-HpaBC, pCS-TPTA
Q10 or R10	pETM6-*Rg*TAL^syn^-HpaBC
Q11 or R11	pXPA-*Rg*TAL^syn^
Q12 or R12	pXPA-*Rg*TAL^syn^, pCS-TPTA
Q13 or R13	pXylA-*Rg*TAL^syn^
Q14 or R14	pXylA-*Rg*TAL^syn^, pCS-TPTA

a “Q” in the strain name indicates strain QH4, while “R” indicates strain rpoA14(DE3).

### Optimization of phenylpropanoic acid production.

**FIG 3:**
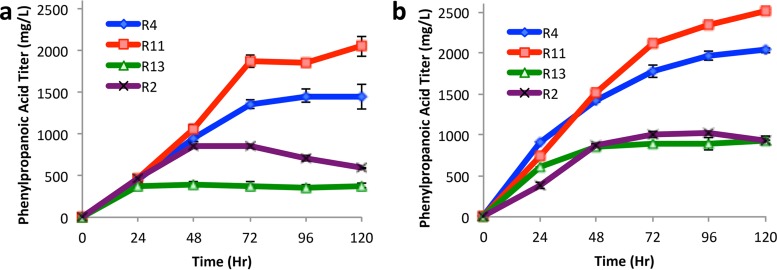
Analysis of top phenylpropanoic acid production modules. (a) Glucose carbon source at 37°C with induction for 3 h (R2 and R4 only). (b) Glycerol carbon source at 37°C with induction for 8 h (R2 and R4 only).

Three *p*-coumaric acid production strains (R4, R11, and R13) and one caffeic acid production strain (R2) from the initial screen were subjected to further optimization to determine the full potential of these modules in monoculture. Through course optimization of the induction point, inducer concentration, production temperature, and carbon source, the highest-titer production to date was realized for both *p*-coumaric and caffeic acids at 2.51 ± 0.03 and 1.03 ± 0.02 g/liter, respectively (Fig. 3). The production of *p*-coumaric acid was found to be highly sensitive to nearly all optimization parameters, with the highest titer achieved using glycerol as a primary carbon source (Fig. 3). Interestingly, caffeic acid production by strain R2 was found to be relatively insensitive to all factors. The titers presented here represent 157% and 34% improvements for *p*-coumaric and caffeic acid, respectively, over the highest titers reported in the literature to date (18, 19). Significant black coloration, indicative of polymeric melanin formation through the l-3,4-dihydroxyphyenylalanine (l-DOPA) intermediate, was observed from all caffeic production modules both on solid as well as in liquid media. This observation is consistent with current literature and is assumed to be the primary factor limiting caffeic acid production (26, 27). Future efforts to scale up to fed-batch fermentation are under way to further improve phenylpropanoic acid titers, yields, and productivity.

### Production of flavan-3-ols *de novo.*

**FIG 4:**
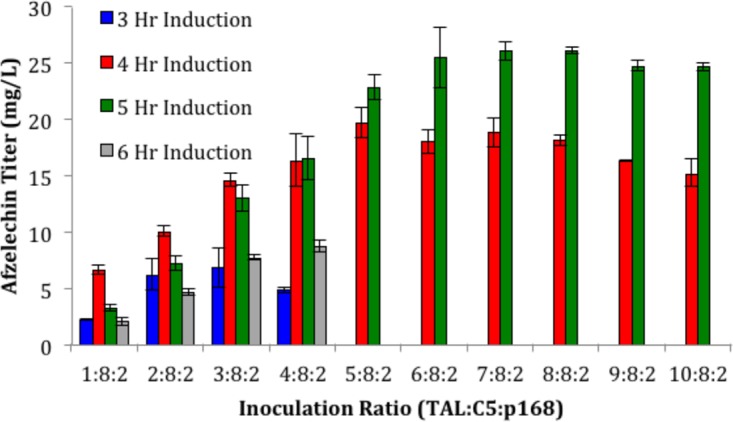
Production landscape of three-strain polyculture for the *de novo* production of (+)-afzelechin. All data were obtained in AMM-glucose medium at a production temperature of 30°C. Error bars represent 1 standard deviation from at least biological duplicate.

Combining the previously published coculture system for the efficient production of flavan-3-ols from phenylpropanoic acids with the best phenylpropanoic acid production module described above enables the production of flavan-3-ols from glucose. Highlighting the drop-in modularity of polyculture systems, we conserved the previously optimized ratio of C5 to p168 of 8:2 (9) and varied only the proportion of the TAL module over several induction points in the range of the predicted optimum from previous work. Using this optimization strategy, we were able to demonstrate the *de novo* production of afzelechin for the first time in a microbial host (Fig. 4). Furthermore, we achieved production titers of 26.1 ± 0.8 mg/liter without extensive optimization. These successes supported the further expansion of flavonoid production using the polyculture platform.

### Production of anthocyanidin-3-*O-*glucosides *de novo.*

**FIG 5:**
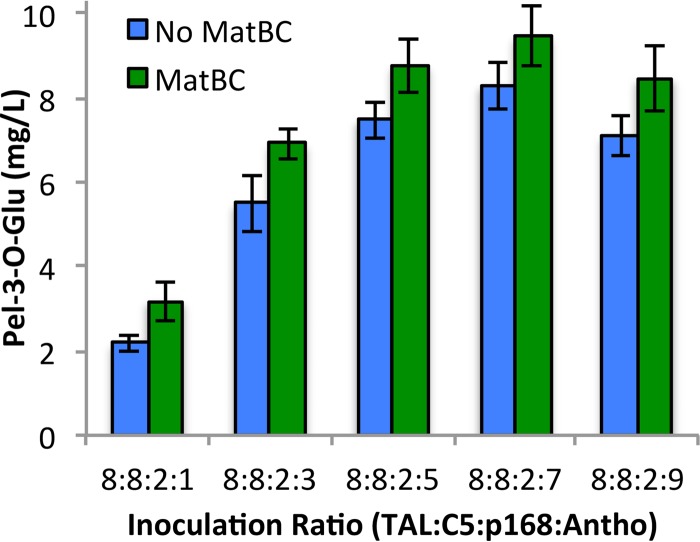
Production of anthocyanidin-3-*O*-glucosides from glucose using a four-strain polyculture. All data were obtained using a 5-h induction point and 30°C induction temperature. Error bars represent ±1 standard deviation from the mean of biological quadruplicates. All differences between MatBC and no-MatBC pairs are statistically significant (*P* < 0.05).

Our previous successes using polycultures for the production of flavonoids motivated the further application of this technology to expand what is currently possible *in vivo*. Previous efforts in our lab have developed strains capable of high-titer anthocyanidin-3-*O-*glucoside production from flavan-3-ols, but efforts to further connect the pathway with the upstream phenylpropanoic acid precursors have not been successful. Building off of these efforts, we selected the previously characterized pETM6-3GT-ANS plasmid for the expression of our flavan-3-ol–to–anthocyanin bioconversion pathway (28). Transformation of this plasmid into our baseline host BL21star(DE3) resulted in our “Antho” module to be combined with the previously described TAL, C5, and p168 modules for the *de novo* production of anthocyanidin-3-*O-*glucosides *in vivo*. In a similar fashion as before, the previously determined optimum ratio of 8:8:2 TAL to C5 to p168) was conserved, with the fraction of the new module being varied to result in the first account of a functional synthetic four-strain polyculture. This microbial consortium enabled, for the first time outside of plants, the production of the anthocyanidin-3-*O-*glucoside callistephin from glucose (Fig. 5).

Addition of two additional enzyme overexpressions, MatBC, to the previously published C5 module further highlights the flexibility of the polyculture platform for rapid expansion and modification. These enzymes enable the uptake of externally supplemented malonate and subsequent activation to malonyl coenzyme A (malonyl-CoA), a key and limiting substrate for the chalcone synthase (CHS) enzyme (29). Significantly higher production (*P* < 0.05) of callistephin from glucose was achieved across a wide range of inoculation ratios, while conserving the optimum fermentation conditions from previous experiments.

## DISCUSSION

The rapid success of polycultures to realize the *de novo* production of various late-pathway flavonoid metabolites demonstrates the power of such approaches over traditional monoculture metabolic engineering efforts (30, 31). Additionally, the ease with which these pathways were reoptimized through conservation of the previously optimized inoculation ratio further highlights the benefits of polyculture modularity over that of traditional monoculture techniques (31). In traditional monoculture techniques, extension of the current heterologous overexpression pathway would require additional genes to be cloned and expressed in the previously optimized strain, consequently deoptimizing the strain from both a genetic and fermentation perspective. Genetic reoptimization is a cumbersome task. Oftentimes, it is impossible to regain the fluxes previously achieved due to either increased metabolic burden (32) or altered natural precursor and cofactor availability, thus limiting the overall titer, yield, and productivity of the process. Polycultures, however, enable the genetic optimization of each module to be preserved, requiring only minor fermentation optimization to adjust the inoculation ratio of the new strain (14). The simplicity of this optimization and the smooth trends observed in corresponding production landscapes support the hypothesis that these cultures are relatively stable through the production phase of the fermentation.

These positive polyculture attributes, however, are not gained without associated costs. The feasibility of our polyculture system hinges on the ability of the intermodule metabolites to be efficiently transported between strains. In the present study, no transporter engineering was required to achieve a functioning system. Careful selection of the pathway division points (e.g., avoiding CoA intermediates as intermodule metabolites) is key to minimizing difficulty in this area. We do envision future applications, especially with products that are membrane associated or lipophilic, where significant effort will need to be performed to ensure transport to and from strain modules. Additionally, in systems where transport must be engineered, this limitation can also be used as an advantage to limit substrate availability to promiscuous enzymes in off-target strain modules.

We have shown high sensitivity to small (less than 5%) changes in initial community composition, which readily facilitates fine-tuning of consortium productivity but could also lead to many unknowns upon scale-up to fed-batch, high-cell-density cultures. Additionally, as subsequent microbial modules are appended to the polyculture, its further expansion is limited by a fixed total biomass to be distributed across all modules. This shortcoming of polyculture-based strategies is minimized when an obvious rate-limiting module is identified, but it can be more pronounced when multiple limiting/bottlenecking modules exist and deplete space available for the more efficient modules in the pathway. Despite these limitations, we have demonstrated the ability of our polyculture-based approach to outperform previously reported efforts to enable the *de novo* production of high-value specialty flavonoids and anthocyanin chemicals.

### Conclusion.

In conclusion, we have constructed and demonstrated the scalability of a high-titer phenylpropanoic acid module that has potential for commercial value if scaled further to a bioreactor. Utilizing this module along with the previously published modules (C5 and p168), we demonstrate the *de novo* production of flavan-3-ols for the first time outside of the native plant hosts. Further expanding on this polyculture theme, we incorporated a fourth module (Antho) containing the anthocyanadin synthase (ANS) and 3-*O*-glycosyltransferase (3GT) genes. Using all four modules, we also showcased, for the first time outside of plants, the production of the anthocyanidin-3-*O-*glucoside callistephin from glucose. This complicated feat was facilitated by the modularity of the polyculture platform, which conserves the genetic optimization of each module and only requires basic fermentation optimization to achieve peak production. The use of this polyculture-based approach has enabled a system complexity only rivaled by a few other success stories in the metabolic engineering literature (33, 34). Our overexpression of 15 unique heterologous or modified (feedback-resistant) enzymes has enabled the *de novo* production of anthocyanins at titers several orders of magnitude higher than those in studies with similar numbers of overexpressed enzymes sourced from eukaryotic organisms. Additionally, the consortium-based approach presented in this proof-of-principle study is not limited to flavonoids but in principle could be applied to a variety of other high-value natural and synthetic products. In summary, coculture and polyculture techniques have demonstrated their potential to rapidly expand what is deemed to be possible with metabolic engineering, but this power comes with additional complexities that must be systematically addressed to achieve the highest titers, yields, and productivities possible.

## MATERIALS AND METHODS

### Bacterial strains, vectors, and media.

*E. coli* DH5α was used to propagate all plasmids. BL21star(DE3), BL21star(DE3)Δ*sucC* Δ*fumC* (35), rpoA14(DE3) (20), and QH4 (18) were used as the hosts for flavonoid production. The expression vectors pETM6 and pXPA were the basis for all plasmid construction and pathway expression. Luria broth (LB) Lennox modification (Sigma) and Andrew’s Magic medium (AMM) (36) were used where noted. All plasmid constructs will be made available through addgene.org.

### Plasmid construction.

Preexisting flavonoid modules were used directly or with slight modification for this work. All plasmids used are summarized in Table S1 in the supplemental material, and all plasmid modifications are described below. Site-directed mutagenesis was performed to silently remove an internal NdeI restriction site from the open reading frame of *Rhodotorula glutinis* tyrosine ammonia lyase (*Rg*TAL^syn^) on pTrc-*Rg*TAL^syn^ (20) using standard methods and primers 3 to 4 (see Table S2 in the supplemental material). The mutagenized *Rg*TAL^syn^ was PCR amplified from pTrc-*Rg*TAL^syn^ using primers 1 to 2 (Table S2). The resulting PCR product was digested (FastDigest; Thermo Scientific) with NdeI and SpeI, gel purified (EZNA MicroElute gel extraction kit; Omega Bio-Tek), and ligated with pETM6 backbone also digested with NdeI and SpeI and gel extracted using standard methods to create pETM6-*Rg*TAL^syn^ (no. 10 in Table S1). The corresponding plasmid was sequence verified (Genewiz, Inc.) and used together with pETM6-HpaBC (no. 12 in Table S1) (22) to create pETM6-*Rg*TAL^syn^-HpaB-HpaC through standard ePathBrick cloning protocols (37).

10.1128/mBio.00621-17.3 TABLE S1  Strains and plasmids used in this study. Download TABLE S1, DOCX file, 0.1 MB. Copyright © 2017 Jones et al. 2017 Jones et al. This content is distributed under the terms of the Creative Commons Attribution 4.0 International license.

10.1128/mBio.00621-17.4 TABLE S2  Primers used in this study. Download TABLE S2, DOCX file, 0.04 MB. Copyright © 2017 Jones et al. 2017 Jones et al. This content is distributed under the terms of the Creative Commons Attribution 4.0 International license.

We replaced the T7-lac feature on pETM6 with the P_xylA_ promoter from *Bacillus megaterium* found on the commercial vector pMM1522 (Mobitec) to create the constitutive expression plasmid pXylA. The PxylA promoter, although xylose inducible in *B. megaterium*, was shown previously to be constitutively expressed in *E. coli* (38). To this end, a gBlock (Integrated DNA Technologies, Inc. [sequence available in Text S1 in the supplemental material]) was synthesized containing the multiple-cloning site (MCS) of pETM6 under the control of the P_xylA_ promoter sequence, flanked by AvrII and SpeI restriction sites on the 5′ and 3′ ends, respectively. The P_xylA_ fragment was then cloned into pETM6 and sequence verified. Two constitutive TAL expression plasmids were obtained by subcloning *Rg*TAL^syn^ from pETM6-*Rg*TAL^syn^ into pXylA and replacing enhanced green fluorescent protein (eGFP) in pXPA-FapO-eGFP (P_GAP_ promoter) (39) at restriction sites NdeI and SpeI using standard methods.

10.1128/mBio.00621-17.1 TEXT S1  gBlock sequence for cloning pXylA. Download TEXT S1, DOCX file, 0.05 MB. Copyright © 2017 Jones et al. 2017 Jones et al. This content is distributed under the terms of the Creative Commons Attribution 4.0 International license.

### Fermentation protocol.

The small-scale cultivation protocol was adapted from Jones et al. (9) with only minor modification. Except where noted, the cultures were grown in AMM with 20 g/liter glucose as the primary carbon source. The cultures were first grown at 37°C and transitioned to 30°C upon induction with 1 mM IPTG (isopropyl-β-d-thiogalactopyranoside). In the case of the phenylpropanoic acid production strains, 125-ml nonbaffled shake flasks containing 25 ml of medium were used to confirm small-scale screening studies, allow for more frequent sampling, and limit evaporation effects on final titer.

### Metabolite analysis.

Analysis methods were slightly adapted from Jones et al. (9). A 25-μl injection was used for all polyculture fermentations. Analysis of phenylpropanoic acid titers in monoculture required a 10-fold dilution of culture broth and a 5-μl injection volume to reach the linear region for UV detection. Absorbance at 280 nm was monitored in all cases, except for anthocyanidin-3-*O-*glucosides, where 518 nm was used. A representative high-performance liquid chromatography (HPLC) chromatogram for anthocyanin quantification is shown in Fig. S1 in the supplemental material. Product titers were determined using authentic standards, while (+)-afzelechin was quantified using the (+)-catechin standard curve in accordance with previous literature, because (+)-afzelechin is not commercially available (22, 40). All experiments were performed in at least biological duplicate, with key high-titer conditions reproduced in biological and experimental triplicate. Error bars represent ±1 standard deviation from the mean. Significance of data was determined using a two-tailed unpaired *t* test with a 95% confidence interval.

10.1128/mBio.00621-17.2 FIG S1  HPLC chromatogram for callistephin quantification. Traces for the commercially available standard (Std) and 4-strain polyculture supernatant are shown. Download FIG S1, PDF file, 0.2 MB. Copyright © 2017 Jones et al. 2017 Jones et al. This content is distributed under the terms of the Creative Commons Attribution 4.0 International license.
