# Supplementary material for: Complete Biosynthesis of Anthocyanins Using E. coli Polycultures
Source: mBio. 2017 Jun 6;8(3):e00621-17. doi: 10.1128/mBio.00621-17 (PMC5461408; doi:10.1128/mBio.00621-17)
Supplement: TABLE S1 [file mbo003173344st1.docx]

Table S1. Strains and plasmids used in this study.

| **Number** | **Strain or vector** | **Relevant properties** | **Reference** |
| --- | --- | --- | --- |
| S1 | *Escherichia coli* DH5α | F^−^, φ80d lacZΔM15, Δ(lacZYA-argF)U169, recA1, endA1, hsdR17(rk^−^, mk^+^), phoA, supE44λ^−^, thi^−1^, gyrA96, relA1 | Novagen |
| S2 | *E.coli* BL21 Star™ (DE3) | F^−^*ompT gal dcm rne131 lon hsdS*_B_ (r_B_^_^m_B_^_^) λ(DE3) | Invitrogen |
| S3 | BLΔsumCΔfumC | BL21StarTM(DE3)ΔfumC::FRTΔsucC::FRT | (35) |
| S4 | rpoA14(DE3) | *E. coli* K12 Δ*pheA* Δ*tyrR lacZ*::P_LtetO-1_-*tyrA*^fbr^*aroG*^fbr^*tyrR*::P_LtetO-1_-*tyrA*^fbr^*aroG*^fbr^ *hisH*(L82R) pHACM-*rpoA*14, λ(DE3) | (20) |
| S5 | QH4 | *E. coli* ATCC 31884/ Δ*pheLA-tyrA* | (18) |
| 1 | pETM6 | ePathBrick expression vector, ColE1 ori, AmpR | (37) |
| 2 | p168 | #1 with CsF3H^syn^-FaDFR^syn^-DuLAR^syn^, monocistronic form | (40) |
| 3 | pETM6-mCherry | #1 with mCherry fluoroscent reporter | (37) |
| 4 | pFlavo^opt^ or C5 mutant | #17 with C4 mutant T7 promoter controlling CmCHI | (9) |
| 5 | pTrc-RgTAL^syn^ | pTrcHis2B carrying codon-optimized *R. glutinis* TAL | (20) |
| 6 | pCS-TPTA | From pCS27, P_L_lacO1; *tyrA^fbr^-ppsA-tktA-aroG^fbr^* | (24) |
| 7 | pZE-TH2 | From pZE12, dual operons, P_L_lacO1; *RgTAL and EcHpaBC* | (18) |
| 8 | pCA1 | pTrcHis2B carrying codon-optimized R. glutinis TAL | (25) |
| 9 | pCA3 | pCDFDuet-1 carrying codon-optimized R. glutinis TAL with a trc promoter | (25) |
| 10 | pETM6-RgTAL^syn^ | #1 with RgTALsyn | This Study |
| 11 | pETM6-RgTALsyn-HpaBC | #1 with RgTALsyn, HpaB, and HpaC in monocistronic form | This Study |
| 12 | pETM6-HpaBC | #1 with HpaB and HpaC in monocistronic form | (22) |
| 13 | pXylA | #1 with constitutive PxylA promoter | This Study |
| 14 | pXPA-fapO-eGFP | pGAP promoter, rrnB terminator and ePathBrick feature carrying one copy of fapO and eGFP | (39) |
| 15 | pXylA-RgTAL^syn^ | #13 carrying RgTAL^syn^ | This Study |
| 16 | pXPA-fapO-RgTAL^syn^ | #14 carrying RgTAL^syn^ | This Study |
| 17 | pMM1522 | Amp^R^ (*E. coli*), Tet^R^ (*B. meg*), pBR322 ori, P_xylA_ | Mobitec |
| 18 | pETM6-At3GT | #1 with 3GT from *A. thaliana* | (28) |
| 19 | pETM6-PhANS | #1 with ANS from *P. hybrida* | (28) |
| 20 | pETM6-At3GT-PhANS | #1 with *At*3GT and *Ph*ANS, monocistronic | (28) |
| 21 | pACYC-matBC | pACYCDuet-1 carrying *R. trifolii* MatB and MatC | (29) |

References:

9. Jones JA, Vernacchio VR, Sinkoe AL, Collins SM, Ibrahim MHA, Lachance DM, Hahn J, Koffas MAG. 2016. Experimental and computational optimization of an Escherichia coli co-culture for the efficient production of flavonoids. Metab Eng 35:55–63.

18. Huang Q, Lin Y, Yan Y. 2013. Caffeic acid production enhancement by engineering a phenylalanine over-producing Escherichia coli strain. Biotechnol Bioeng 110:3188–3196.

20. Santos CNS, Koffas M, Stephanopoulos G. 2011. Optimization of a heterologous pathway for the production of flavonoids from glucose. Metab Eng 13:392–400.

22. Jones JA, Collins SM, Lachance DM, Vernacchio VR, Koffas MAG. 2016. Optimization of naringenin and p -coumaric acid hydroxylation using the native E. coli hydroxylase complex, HpaBC. Biotechnol Prog 32:21–25.

24. Lin Y, Yan Y. 2012. Biosynthesis of caffeic acid in Escherichia coli using its endogenous hydroxylase complex. Microb Cell Fact 11:1–9.

25. Zhang H, Stephanopoulos G. 2013. Engineering E. coli for caffeic acid biosynthesis from renewable sugars. Appl Microbiol Biotechnol 97:3333–41.

28. Cress BF, Leitz QD, Kim DC, Amore TD, Suzuki JY, Linhardt RJ, Koffas MAG. 2017. CRISPRi-mediated metabolic engineering of E. coli for O-methylated anthocyanin production. Microb Cell Fact 16:10.

29. Leonard E, Yan Y, Fowler ZL, Li Z, Lim C-G, Lim K-H, Koffas MAG. 2008. Strain improvement of recombinant Escherichia coli for efficient production of plant flavonoids. Mol Pharm 5:257–65.

35. Xu P, Ranganathan S, Fowler ZL, Maranas CD, Koffas M a G. 2011. Genome-scale metabolic network modeling results in minimal interventions that cooperatively force carbon flux towards malonyl-CoA. Metab Eng 13:578–87.

37. Xu P, Vansiri A, Bhan N, Koffas MAG. 2012. ePathBrick: A Synthetic Biology Platform for Engineering Metabolic Pathways in E. coli. ACS Synth Biol 1:256–66.

39. Xu P, Li L, Zhang F, Stephanopoulos G, Koffas M. 2014. Improving fatty acids production by engineering dynamic pathway regulation and metabolic control. Proc Natl Acad Sci 111:11299–11304.

40. Zhao S, Jones JA, Lachance DM, Bhan N, Khalidi O, Venkataraman S, Wang Z, Koffas MAG. 2015. Improvement of catechin production in Escherichia coli through combinatorial metabolic engineering. Metab Eng 28:43–53.
