# Supplementary material for: Complete Biosynthesis of Anthocyanins Using E. coli Polycultures
Source: mBio. 2017 Jun 6;8(3):e00621-17. doi: 10.1128/mBio.00621-17 (PMC5461408; doi:10.1128/mBio.00621-17)
Supplement: TABLE S2 [file mbo003173344st2.docx]

Table S2. Primers used in this study.

| **Primer ID** | **Primer Name** | **Sequence (5’→3’)** |
| --- | --- | --- |
| 1 | RgTALsyn_FWD w/NdeI | GCGGCGCATatggcgcctcgcccgacttc |
| 2 | RgTALsyn_REV w/SpeI | GCGGCGACTAGTttatgccagcatcttcagcagaacattg |
| 3 | SDM_RgTALsyn_FWD | Gcactgcacgacgcgcacatgttgagcctgttgagc |
| 4 | SDM_RgTALsyn_REV | Gctcaacaggctcaacatgtgcgcgtcgtgcagtgc |
| 5 | pXylA_FOR | GCAAGCATGCGAAATGCA |
| 6 | pXylA_REV | GAGTTTCGTTCGAGATCGC |
