# Supplementary material for: Complete Biosynthesis of Anthocyanins Using E. coli Polycultures
Source: mBio. 2017 Jun 6;8(3):e00621-17. doi: 10.1128/mBio.00621-17 (PMC5461408; doi:10.1128/mBio.00621-17)
Supplement: TEXT S1 [file mbo003173344s1.docx]

### Sequence S1. gBlock Sequence for cloning pXylA:

GCAAGCATGCGAAATGCACCTAGGAAAAAAAACATTGAAATAAACATTTATTTTGTATATGATGAGATAAAGTTAGTTTATTGGATAAACAAACTAACTCAATTAAGATAGTTGATGGATAAACTTGTTCACTTAAATCAAAGGGGGAAATGTACACATATGGCAGATCTCAATTGGATATCGGCCGGCCACGCGATCGCTGACGTCGGTACCCTCGAGTCTGGTAAAGAAACCGCTGCTGCGAAATTTGAACGCCAGCACATGGACTCGTCTACTAGTCGCAGCTTAATTAAGCGATCTCGAACGAAACTC
