# Supplementary figures and images for: Complete Biosynthesis of Anthocyanins Using E. coli Polycultures
Source: mBio. 2017 Jun 6;8(3):e00621-17. doi: 10.1128/mBio.00621-17 (PMC5461408; doi:10.1128/mBio.00621-17)

DAD1 B, Sig=518,4 Ref=off (ANDREW\2016\FLAVONOIDS 2016-02-26 15-44-12\882A5-1.D)

DAD1 B, Sig=518,4 Ref=off (ANDREW\2016\FLAVONOIDS 2016-02-26 15-44-12\PELAR-3GLUSTD.D)

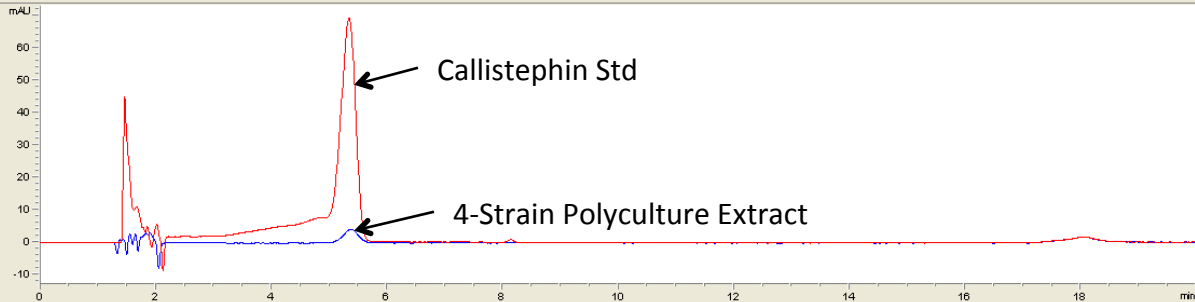

Supplement: FIG S1 [file mbo003173344sf1.pdf]
